# Elasto-mechanical properties of living cells

**DOI:** 10.1016/j.bbrep.2016.06.015

**Published:** 2016-06-22

**Authors:** Béla Varga, Csilla Fazakas, Imola Wilhelm, István A. Krizbai, Zsolt Szegletes, György Váró, Attila G. Végh

**Affiliations:** Institute of Biophysics, Biological Research Centre of the Hungarian Academy of Science, Szeged 6726 Hungary

**Keywords:** Atomic force microscopy, AFM, Young's modulus, Endothelial cell, Quasi-periodic oscillation

## Abstract

The possibility to directly measure the elasticity of living cell has emerged only in the last few decades. In the present study the elastic properties of two cell lines were followed. Both types are widely used as cell barrier models (e.g. blood-brain barrier). During time resolved measurement of the living cell elasticity a continuous quasi-periodic oscillation of the elastic modulus was observed. Fast Fourier transformation of the signals revealed that a very limited number of three to five Fourier terms fitted the signal in the case of human cerebral endothelial cells. In the case of canine kidney epithelial cells more than 8 Fourier terms did not result a good fit. Calculating the correlation between nucleus and periphery of the signals revealed a higher correlation factor for the endothelial cells compared to the epithelial cells.

## Highlights

• Hours long time dependent elasticity measurements were performed on living cells.
• Quasi-periodical oscillation of the elastic modulus was detected with AFM.
• FFT and correlation factor calculation prove the existence of these oscillations.

## 1 Introduction

All living organisms are dynamic systems, driven by well defined and synchronized mechanical processes between different parts of the whole [1], [2], [3]. The smallest living unit, with a complex function, is the cell. Uncountable types of cells, each with different well determined functions can form an organ or organism. To understand the function of a highly complex organism first we have to know its basic unit, the cell. Distinctive role have the barrier forming cells, which separate and link selectively important parts of a body.

Regarding the proper homeostasis of the Central Nervous System (CNS) the importance of the cerebral endothelial cells (CEC) cannot be questioned. They constitute the structural basis of blood-brain barrier (BBB), having crucial role in the control of trafficking substances across their membrane [4], [5] to and from the CNS. While cerebral endothelial cells have a principal role in the maintenance of the homeostasis of the CNS, epithelium of the renal distal tubule contributes to the ion homeostasis of the organism. Although they are intensively studied, still limited information is available about their function, or how their internal molecular alterations manifest in mechanical properties such as in elasticity coefficient or molecular adhesion [6].

A rather new tool for determining mechanical properties, such as the elasticity of a microscopic object, is the atomic force microscope (AFM) [7], [8]. Besides atomic resolution surface topography, the AFM can provide quantification of micro-mechanical parameters as well. A great advantage of it is that the measurements can be performed not only in vacuum, but in air or in liquid environment at human body temperature. The later is indispensable in case of living cells [9], [10].

Mechanical properties of individual cells are strongly connected to biological functions, dynamically linked to both internal and external stimuli. Measuring the time dependence of mechanical properties of a biological system, spontaneous quasi periodical oscillation could be observed. Oscillation can appear in open nonlinear dynamic system. Biological systems fulfill these conditions [1], [11]. The first documented biological oscillation was described by Luigi Galvani in 1780. Just to name few examples when oscillation was observed: mechanical and electrical oscillation in cardiac muscle of the turtle [12] drosophila tissue motion [13], oscillation of the elasticity and adhesion of vascular smooth muscle cell [14], shape oscillations of human neutrophil leukocytes [15], bronchial epithelial cells [16] elasticity oscillation of the cerebral endothelial cells [17]. The period of these oscillations show large variability, spanning from seconds to hours. Although more and more type of cells are intensively studied and several oscillating cells were investigated, apart of induced contractions e.g. muscle cells, the conditions when and why they emerge is mostly undeciphered.

In the present study the elastic oscillation measured on human brain micro-vascular endothelial and canine kidney epithelial cells were investigated and compared. Both cell lines are widely used as models mainly of the Blood-Brain barrier [18]. While the vascular endothelial cells are constantly exposed to mechanical forces from the blood stream the epithelial cells do not have to withstand shear forces. Hence their response to mechanical stimuli may result in completely different manifestation. All these information can help to understand the origin of emerging oscillations.

## 2 Materials and methods

### 2.1 Cell culture

The human cerebral micro-vascular endothelial cells (hCMEC/D3 - shortly D3) were grown on rat tail collagen-coated dishes in EBM-2 medium (Lonza) supplemented with EGM-2 Bullet Kit (Lonza) and 2.5% Fetal Bovine Serum (FBS) from Sigma [19], [20]. MDCK (Madin-Darby canine kidney) cells were maintained in DMEM/F-12 (Dulbecco’s Modified Eagle Medium/Nutrient Mixture F-12) (Lonza) supplemented with 5% FBS.

Cells were cultured at 37 °C, in 5% CO_2_ atmosphere, seeded at 1.5*10^4^ cell/cm^2^ in Falcon Petri dish (lid) with 3.5 cm diameter. MDCK monolayers were fed with fresh medium first after 24 h (post-seeding) than every second day until they reached confluence (3rd day).

All measurements were performed in serum free Leibovitz medium (Sigma) at 37 °C within 3 h after taking the cells out from the incubator. The temperature was kept constant using a home made heating stage at accuracy of 0.1 degree. According to our observations and to literature, in Leibovitz medium cells preserve their viability within this time period [21].

### 2.2 AFM

All experiments were carried out with an Asylum Research MFP-3D atomic force microscope (Asylum Research, Santa Barbara, CA; driving software IgorPro 6.32A, Wavemetrics), mounted on a Zeiss Axiovert 200 optical microscope. The experiments were performed with gold coated silicon nitride rectangular cantilevers, nominal spring constant of 0.03 N/m, resonant frequency 37 kHz in air which drops to 10 kHz in liquid, with a “V” shaped tip (Olympus, Optical Co. Ltd). The spring constant of the cantilever was determined each time by thermal calibration [22]. All images were recorded in Alternate Contact (AC) mode having 256 lines by 256 points, tip velocity of 60 µm/s. Trace and retrace images were both recorded and compared for internal accuracy. Noteworthy differences could not be found which underlines their reliability. On both cell lines more than 20 experiments were run. A representative set is reported in this paper.

### 2.3 Force measurements

**Fig. 1:**
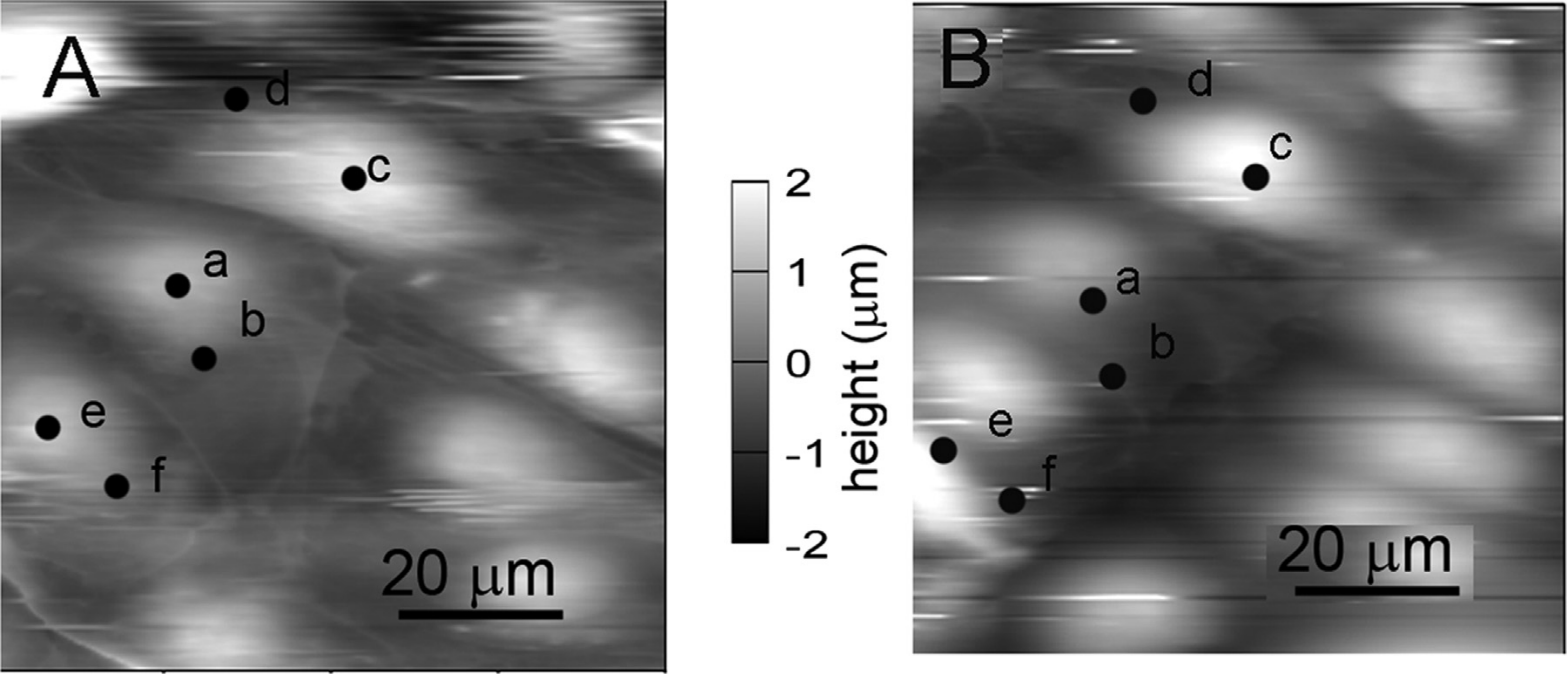
Image of endothelial D3 cells (panel A) before and (panel B) after the force measurements. The dots with letters (a-b-c-d-e-f) show the locations where the forces were measured cyclically.

**Fig. 2:**
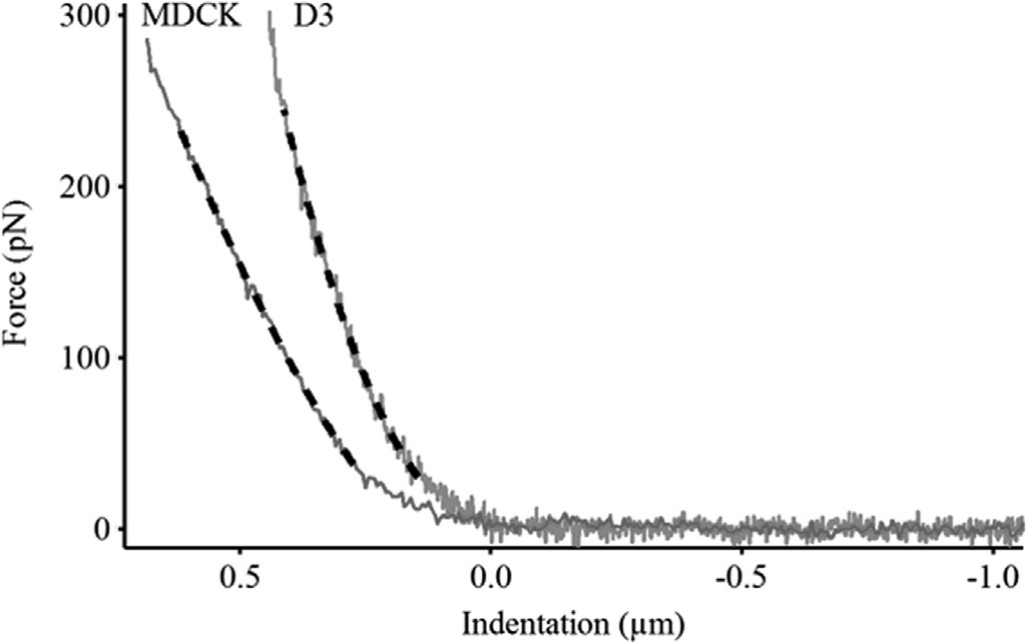
Force versus indentation signal recorded on D3 and MDCK cells, with Hertzian fit (black). Only the approaching curve (trace) is presented.

After taking an image three cells were chosen, with selected points on nuclear and peripheral region respectively (Fig. 1). Force curves were measured consecutively on each pre-defined spot on cells, having equal time elapsed between two consecutive measurement, which was typically 30 s. The force curves were recorded in contact mode at constant loading speed (2 µm/s) and sampling frequency (0.5 kHz). Total force distance was kept at 3 µm and maximum load below 0.3 nN. Through the whole text we use elasticity which is directly measured. A constant multiplication factor could convert in elasticity constant or Young's modulus. Probing any material with a hard indenter (AFM tip) leads to the theory of indenting an elastic half-space with a stiff object, based on the work of Heinrich Hertz [23] and Ian Sneddon [24] later modified for AFM tips [25]. This theory is used for indentation tests, regardless of length scale. Elastic characterization was based on calculating the sample's elastic modulus [17], [26] from each force curve. Fig. 2 shows the Force-Indentation curves recorded on both cell types, and the black lines represent the Hertzian fit. Both the upper and lower 10% of data beyond 0 indentations (contact point) were omitted in order to avoid internal errors of contact point determination and high force induced uncertainty. Data extraction and elasticity calculations were performed with the AFM driving software (IgorPro 6.32A, Wavemetrics).

### 2.4 Data analysis

Periodical oscillations can be described with three different parameters: amplitude, frequency (period) and phase. In our case the phase was neglected, since it is meaningful if a reference time point is available. A home made MatLab (Math Works Inc., Natick, Massachusetts) routine was implemented to calculate the amplitude and frequency spectra of the elastic variations and their reproduction based on sum of sine functions. Discrete Fourier Transform (DFT) method using a Fast Fourier Transform algorithm is a widespread method to quantify and convert periodic signals to frequency domain [27]. The spectra can be represented in function of both frequency and period. In our case the latter being more straightforward. The obtained frequency spectra were smoothed to avoid fragmentation. This does not induce any change in peak position, only makes them easier to be identified. In order to estimate the number of different oscillations that can be found in the recorded signals, a fit with sinusoidal waves was applied to raw data. For fitting sum of sine waves non of their parameters (amplitude, frequency, phase) were kept constant obtaining the fit simultaneously. To characterize the similarities between the two data sets, the Pearson's correlation coefficient was calculated.

## 3 Results

**Fig. 3:**
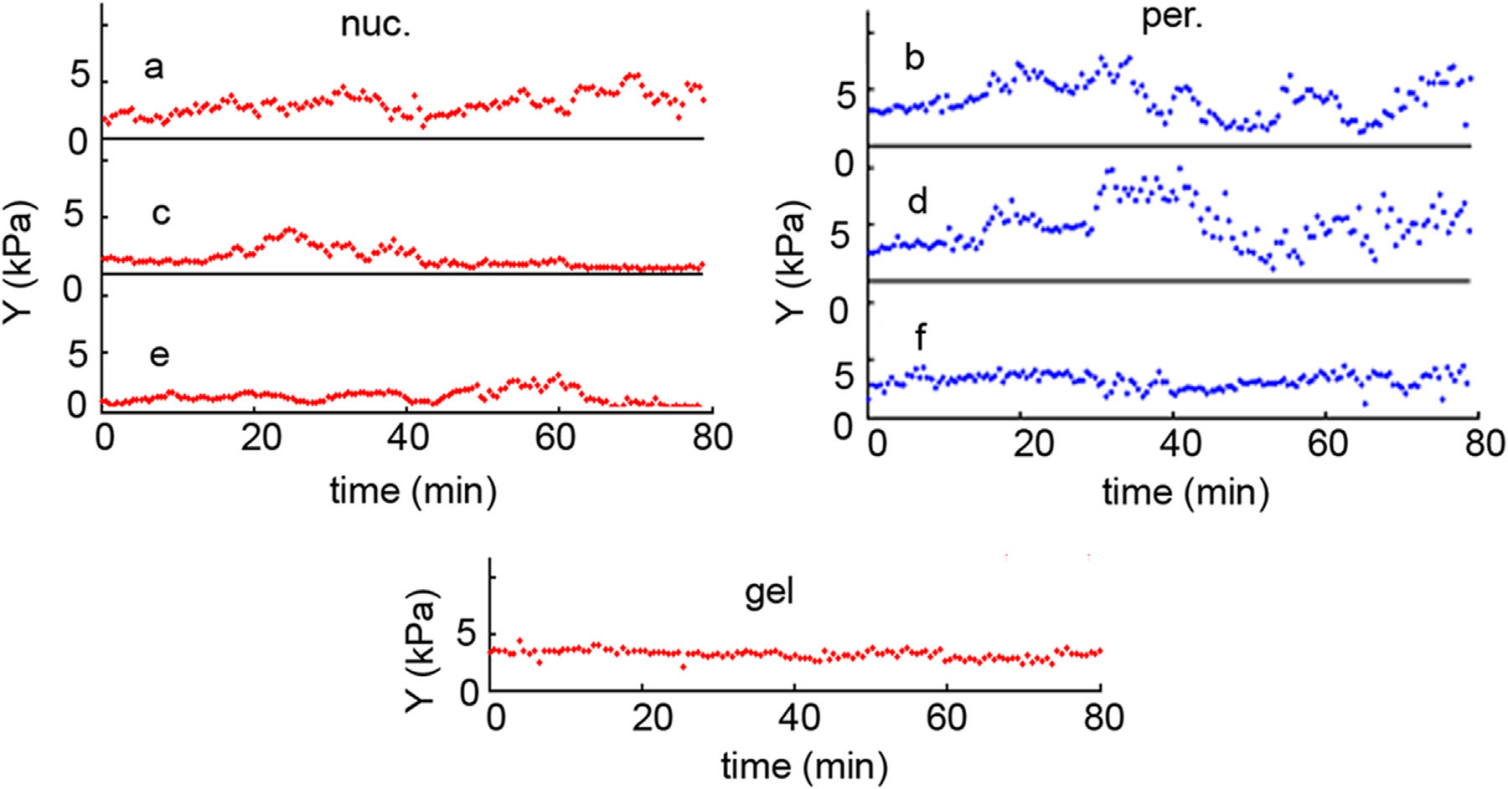
The time dependency of living D3 cell elasticity measured periodically over the nucleus (a, c, e) and at the periphery (b, d, f). The curve “gel” is the control measured on acrylamide gel in a similar manner.

High resolution topographies were made on living cells grown in a Petri dish (lid). In each case three cells were chosen with proper shape. On each cell two different locations were selected: one over the nucleus, the other at the cell periphery (Fig. 1). At these selected six points elasticity measurements were effectuated cyclically. Duration of one cycle was about 30 s. The whole experiment lasted for 60–80 min, resulting 120–150 measurement at each point. During the experiment in each selected place a classical force curve was taken (Fig. 2) and the elasticity of the measured point was calculated. In this way the time dependence of the cell elasticity in the selected points could be followed (Fig. 3) simultaneously.

The size of the noise of the whole system was estimated by replacing the cells in the Petri dish with a thin layer of acrylamide gel and the elasticity on six points was measured in similar conditions. The fluctuation of the time traces is larger than the noise. All six traces were almost straight lines, out of which only one is presented (Fig. 3, curve gel). No fluctuations can be distinguished at similar scale to those on living cells.

**Fig. 4:**
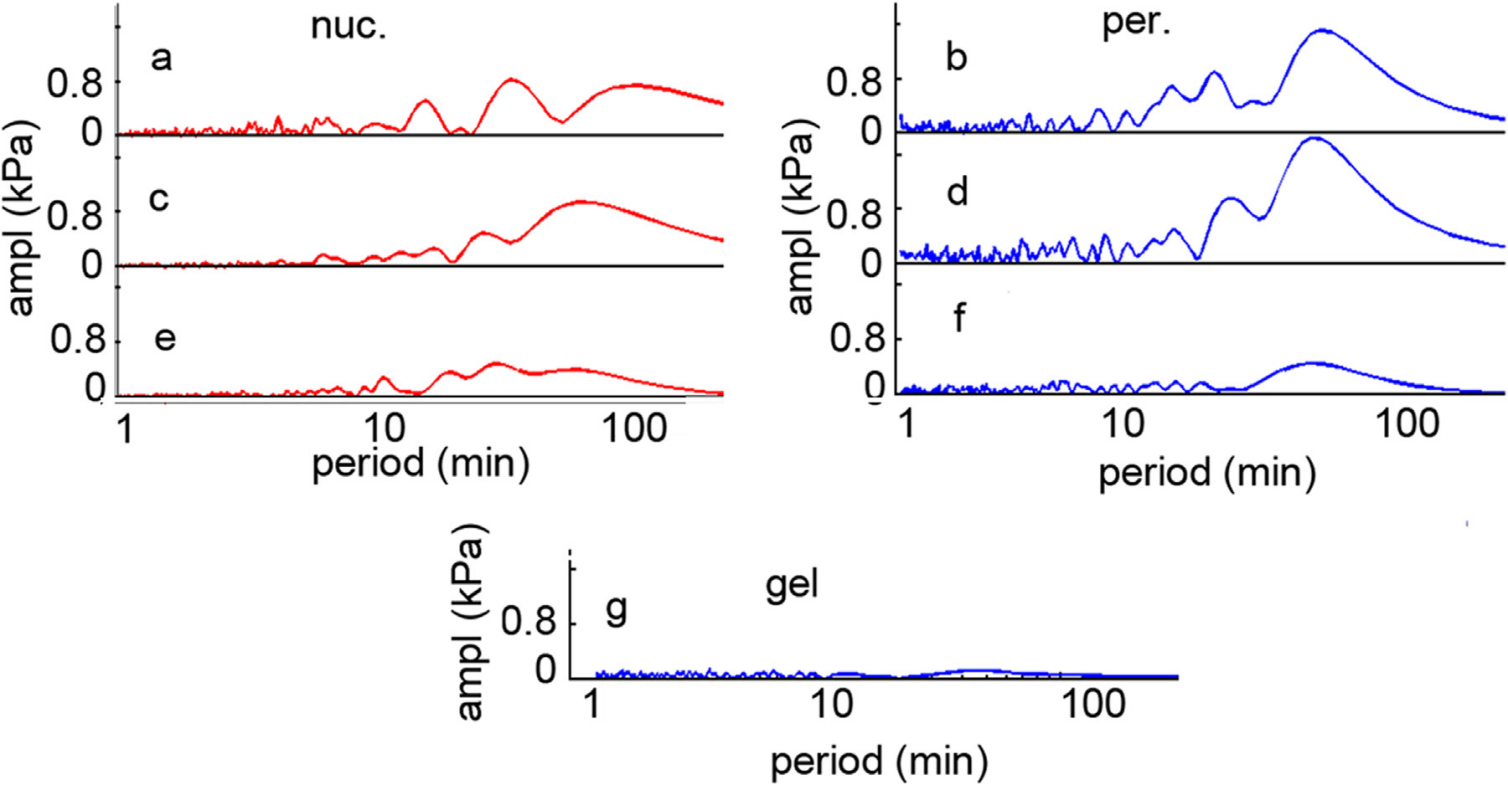
Period domain spectra calculated with Fast Fourier Transformation of the corresponding signals shown on Fig. 3.

The obtained FFT signals hold the period spectra of the calculated elasticity, containing several sinusoidal components with well determined time period. Each peak represents a period of an oscillating sinusoidal wave present in the raw data. To eliminate the very slow shift and the fast noise like component a Fast Fourier Transform (FFT) algorithm was applied on the time dependent elasticity series (Fig. 4) and the periods below 5 min and over 100 min were cut.

**Fig. 5:**
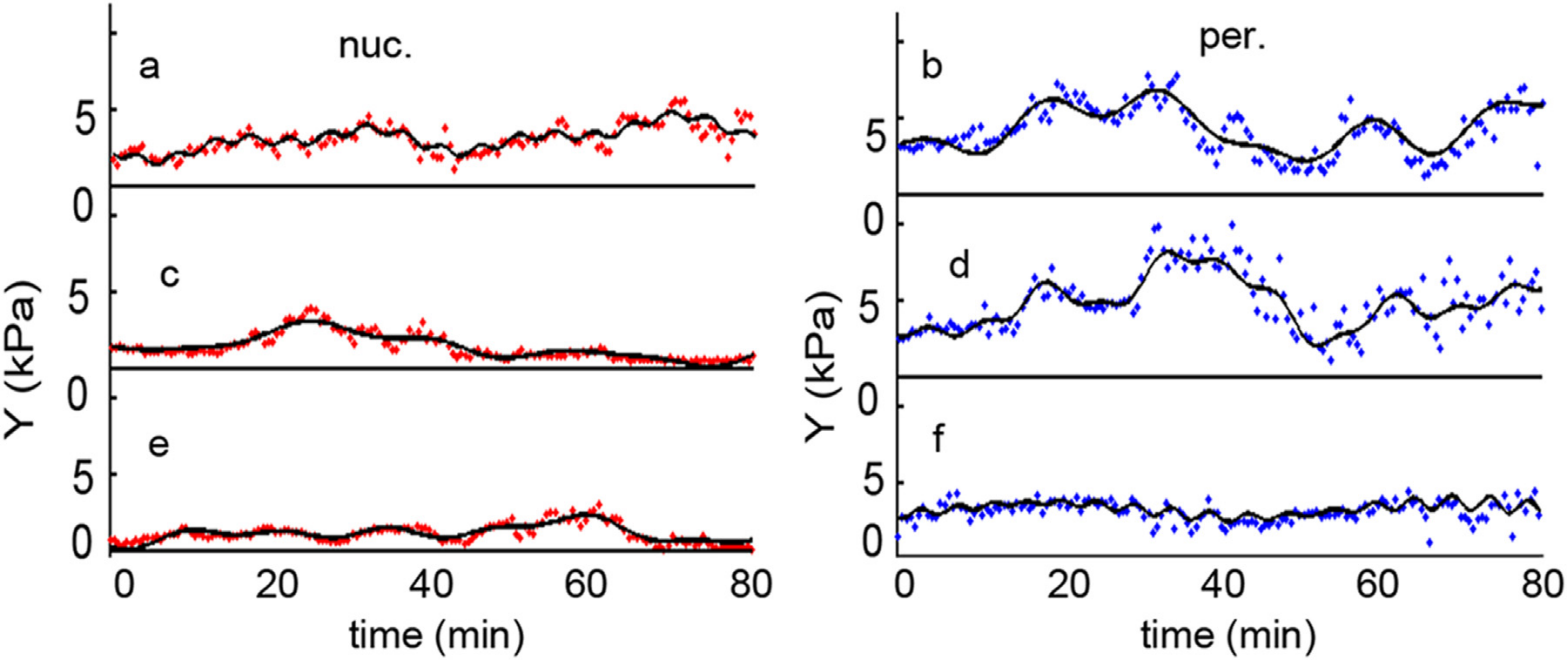
The signals from Fig. 3 (dots) fitted with sum of 4 sine curves (continuous line).

The former was considered too quick to be accurately followed in our system, the latter too slow for proper calculations at this time scale. The truncated curves were converted back to the time space with an Inverse Fast Fourier Transform (IFFT). (Data not shown) This led to a nice noise reduction, still having the main variations of the original curves. In order to estimate how many oscillating components describe the time dependent elasticity traces, the most intense peaks were selected from the FFT spectra. A multi sinusoidal fit was applied to original signal, ranging from one to eight sine components. Our data analysis led to determination of the four most intense periods, which were enough to describe the recorded signal with high accuracy (Fig. 5).

**Fig. 6:**
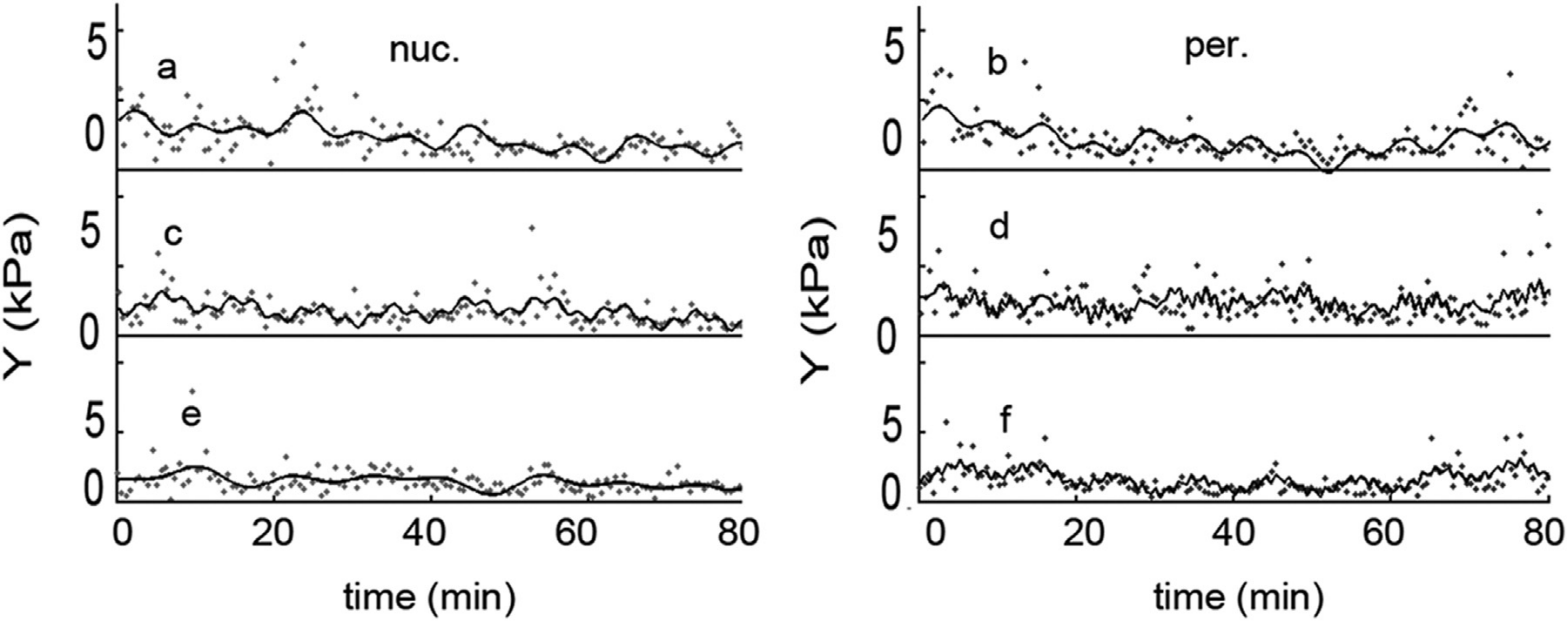
The time dependency of the epithelial MDCK cells elasticity. The measuring and calculation protocols were similar as used for the D3 cells. The dots are the measured signal while the continuous line is the fit with 4 sinus curve.

**Fig. 7:**
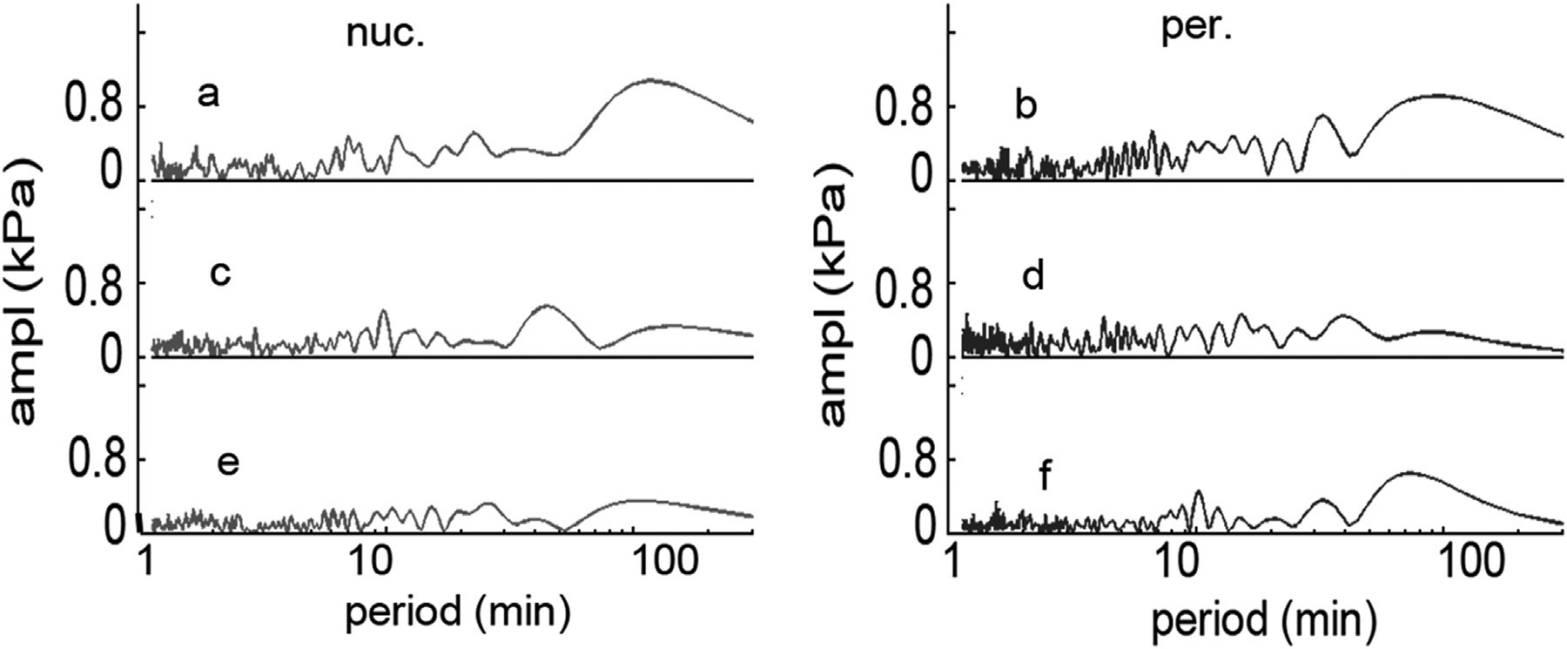
Fast Fourier Transform spectra of the signals shown on Fig. 6.

Browsing through the elasticity series three different kind could be distinguished based on their oscillating amplitude: large amplitude (Fig. 5a, b, d,) small amplitude (Fig. 5 e, f) and transitional traces (Fig. 5c). Similar set of experiment was measured on MDCK epithelial cells and the data treated in a similar mode. The tendency of the elasticity signal was similar to that measured on D3 cells, but the noise was commensurable with the signal (Fig. 6 dots). The data analysis yielded apparently faster FFT components (Fig. 7), with an almost constant amplitude for large time intervals.

**Fig. 8:**
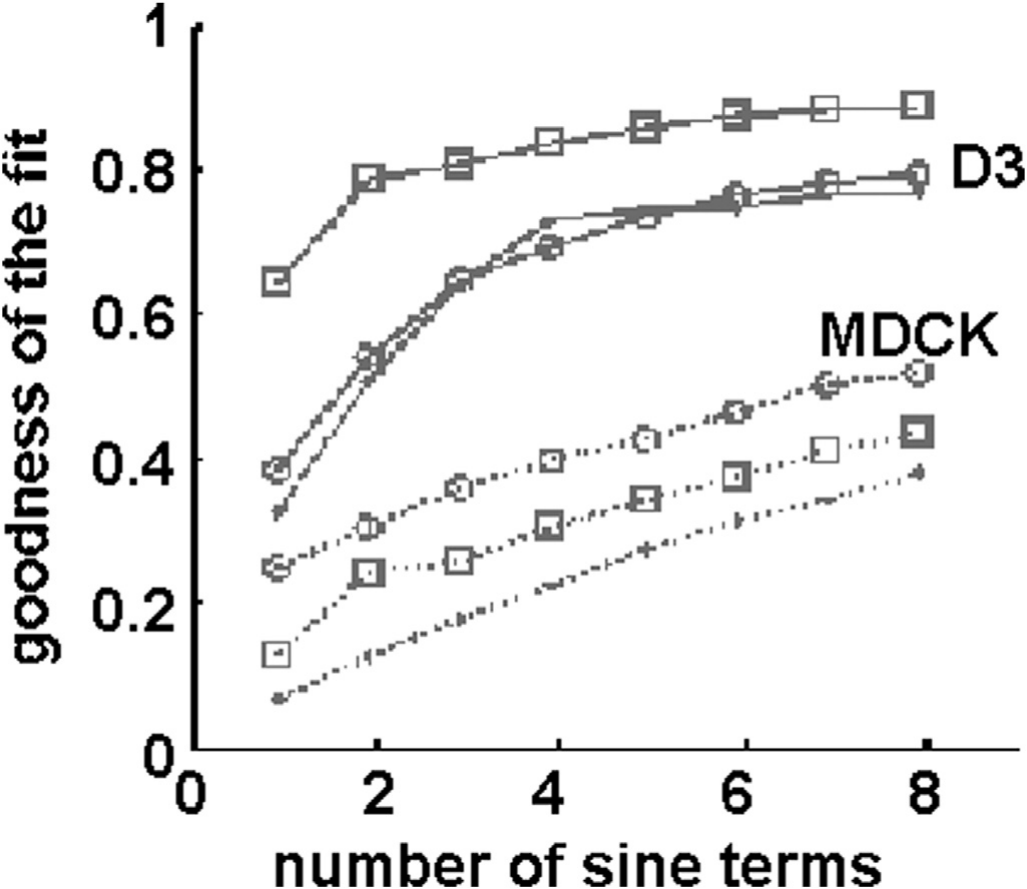
The increase of the goodness of fit with increasing number of sinusoidal.

The fit to the signals belonging to D3 cells resulted a good fit with 4 sinusoidal (Fig. 5, line), further component not improving considerably the fit, which has saturated after adding four components (Fig. 8). Contrary to this the MDCK cells did not saturate even with 8 components and the fit was not improving (Fig. 6, Fig. 8).

**Fig. 9:**
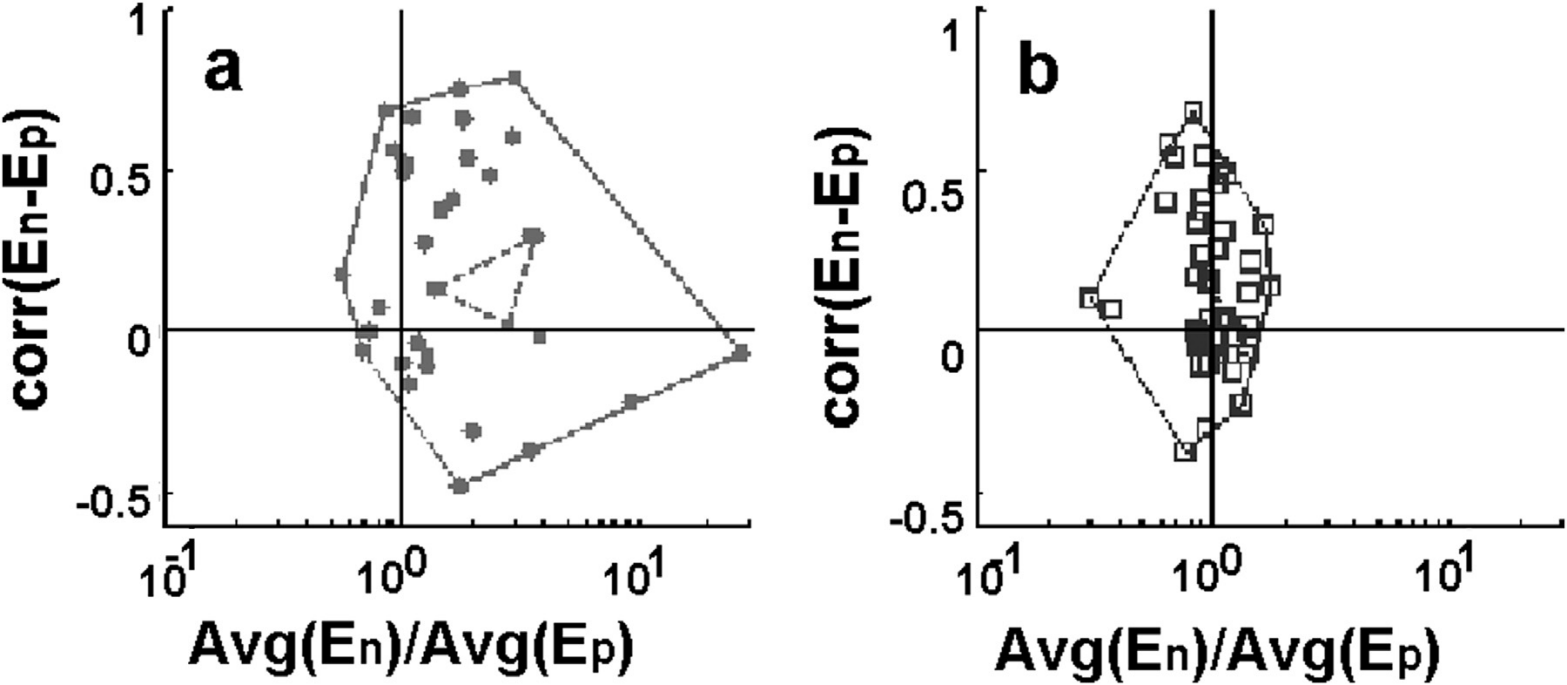
The correlation coefficient between elasticity signals of nuclei and periphery calculated for all measured D3 (a) and MDCK (b) cells versus the average value of each respective signal. Within the convex hull, the values corresponding to the presented data are connected with dashed line.

The next step of the analysis was based on the assumption that interaction might exist between different parts of the cell and this is reflected in a cooperative change of several parameter reflecting the function of the cell. Such a parameter is the elasticity of the cell. To get closer in the analysis of the data to observe the cooperative behavior of the elasticity, the correlation between the time dependent series taken above nucleus and periphery were calculated (Fig. 9).

## 4 Discussion

Oscillations associated with cells as a living object could describe fluctuations from the interior of the cell. Our aim is to develop a model, to describe the changes of the elasticity related to the events happening in the cell. These events are apparently random in time and space. The large number of cells, each receiving and transmitting several signals makes the system complex.

The amplitude of the fluctuation should be related to the activity of the studied part of the cell. If the measured point is close to an active part its elasticity is varying due to molecular structural changes either in the cytoskeleton or in the organelle in the cytosol or in the glycocalyx. This elasticity change produces a “pressure shock” which propagates in the cytoplasm and in the extracellular medium, producing a signal for the neighboring active part. The signal can influence an active part positively by more activation or negatively, by inhibiting it, depending in the earlier state and other signal arriving in the same time. Unfortunately, due to the nature of the measurement, no independent measure is available to monitor surface or size changes separately. Although these alterations cannot be excluded, as the two different cell types were treated in the same manner, their differences are real and still comparable.

The specially chosen sequence of measurement gives possibility of comparing the series recorded in the same time interval at the same different locations (Fig. 1). The quasi-oscillations show a large variety of amplitude and frequency on the endothelial cell (Fig. 3). As a control a thin layer of acrylamide, as a mostly elastic material, was measured in similar sequential mode. No change in the elasticity could be observed. Another control was published earlier which proved that the oscillation is related to the living cell [17]. Although in that case the total monitoring period was a bit shorter, after fixation the cell showed only noise in the time dependent elasticity signal. Both controls prove that the measured elasticity signal originates from the living cell.

The recorded elasticity traces were mathematically processed. The FFT decomposed signal was truncated at the long period end which corresponds to a baseline shift, with still unknown origins. The other end, which contains the noise was also cut (Fig. 4). The FFT spectrums of the endothelial cells are dominated by several long lifetime components, of which several can be clearly distinguished. The epithelial cells contain more components with almost identical amplitude. The result was reconverted back with inverse FFT resulting a filtered signal. Furthermore, the original elasticity signal was fitted with increasing number of sinus curves, in order to find how many components can be clearly distinguished.

The correlation between the series was compared in case of nuclei and their peripheral counterparts. By plotting the calculated correlation factor in function of elasticity ratio, it was obtained an asymmetric arrangement of the points with average value for D3 cells 0.23 while for the MDCK cells this value was only 0.12. A much smaller value as it was predicted by the sinus fit of the signals. The correlation of the elasticity of the D3 cells were larger compared to the MDCK cells. This might point towards that endothelial cells have higher density of cytoskeletal network filaments at peripheral regions, while epithelial cells seem to be more homogenous in this manner.

All these analysis show that a characteristic difference exists between the endothelial and epithelial cell mechanical properties. While the average value of the elasticity is almost the same, the oscillation of the two cell types are different in frequency and amplitude. These might be related to dynamics related to outer layer of the glycocalyx, cell membrane and cytoskeletal components. Unfortunately, our model cannot resolve at this time point the exact link to each component, but further experiments may contribute to their investigation.
